# Enhancing road safety of PLEVs by novel vehicle concepts: A comprehensive investigation on regulations, accident statistics, and perception of riders in Europe

**DOI:** 10.1016/j.heliyon.2024.e41129

**Published:** 2024-12-13

**Authors:** Michelangelo-Santo Gulino, Susanna Papini, Giovanni Zonfrillo, Peter Miklis, Thomas Unger, Dario Vangi

**Affiliations:** a Department of Industrial Engineering, Università degli Studi di Firenze, Via di Santa Marta, 3, 50139, Firenze, Italy; b Verkehrsunfallforschung an der TU Dresden Gmbh, Semperstraße 2a, 01069 Dresden, Germany

## Abstract

The rise of Personal Light Electric Vehicles (PLEVs), including electric bicycles and electric kick scooters, represents a relevant trend in current urban mobility. PLEVs offer economic, social, and environmental advantages, making them increasingly attractive for short-distance travel. Despite their benefits, concerns about the safety of PLEVs, particularly related to road accidents, have arisen due to their growing presence in urban areas.

This paper examines various aspects of PLEV safety, focusing on equipment required by regulations, circulation aspects, and users' needs; based on such highlights, the objective is to derive a vehicle concept (in the form of a kick scooter) that integrates elements for PLEV safety maximization starting from the design phase. A review of PLEV-related regulation in European countries, and particularly for electric kick scooters, is initially provided to determine the mandatory equipment for their circulation in the widest possible number of countries. Analysis of accident data for PLEVs in the EU then reveals critical factors contributing to the occurrence of crashes, injuries, and casualties, as well as the type and severity of riders' injuries. Surveys on user perceptions shed light on safety concerns of daily riders, indicating the most relevant aspects to enhance from the user's perspective.

Based on these findings, the study proposes a novel electric kick scooter concept integrating safety features derived from regulations, accident analysis, and user needs. Safety equipment aimed at increasing riding stability (high diameter wheel, pneumatic tyres) appears as the most promising solution for maximizing PLEV safety. Inclusion of turning light indicators can also increase riding stability, since the user can exhibit his/her intention of turning while maintaining the contact with the handlebar; as of today, this latter feature is mandatory only in Italy among all EU countries. The work additionally emphasizes the need for harmonized regulations, enhanced safety standards, specifically designed infrastructure, and user awareness campaigns to improve PLEV safety. This research is functional for the proposal of a sustainable and safe transportation solution that can increase the consensus towards PLEVs and the consequent spread of electric microvehicles.

## 1 Introduction

The increasing number of electric microvehicles in the form of Personal Light Electric Vehicles (PLEV), such as powered bicycles, several types of e-kick scooters and powered boards, represents a significant trend in contemporary urban mobility. These vehicles offer a range of economic, social, and environmental advantages that make them increasingly attractive to users and authorities seeking sustainable and inclusive urban mobility solutions [1], [2], [3], [4], [5]. PLEVs are characterized by their lightweight construction, battery-powered electric motors, and relatively low speeds compared to traditional motor vehicles. They provide a convenient and environmentally friendly alternative for covering short distances, contributing to reduced emissions and congestion in the cities [6], [7]. In particular, e-kick scooters have gained significant popularity in recent years due to their convenience, ease of use, and versatility in urban areas. In Europe, there are approximately 20 million e-scooter users, and projections suggest that the micromobility market will exceed a value of 100 billion Euros by 2030. For comparison, the entire car-sharing market in Europe was valued at just half a billion Euros back in 2017, according to a 2020 study by the EU Urban Mobility Observatory.^1^ They can be easily rented via mobile apps and used for short trips within the city. E-kick scooters offer a quick and efficient alternative to traditional modes of transportation and are particularly suitable for last-mile travels. Numerous studies focus on integrated mobility with PLEVs in various scenarios and contexts [8]. For instance, by the initial evolution of shared micromobility in New Zealand cities where shared e-kick scooters started circulating in 2018, the complexity of this new transportation practice is evident, influenced by factors such as existing infrastructure, regulatory interventions, and changing perceptions of space use legitimacy [9]. For evaluating travel behaviour on e-kick scooters, surveys were conducted in pilot cities such as Barcelona, Munich, Copenhagen, Tel Aviv, and Stockholm to examine the services provided, customer satisfaction, travel combinations, and regulations concerning e-micromobility [10]. The strengths and weaknesses of this type of mobility and its future implications were highlighted. Among the identified issues are unsuitable infrastructures, conflicts with other road users, and the need for preferred or dedicated infrastructure leading to safer usage; another significant aspect is the user's lack of awareness of specific laws regarding microvehicles. A comprehensive analysis regarding travel behaviours of e-kick scooter riders is provided in the scoping review by Janikian et al. [11]: studies between 2017 and 2022 focusing on safety issues related to dockless e-kick scooters highlight underage riding, double riding, speeding, and rider distraction as the most relevant riders' risky behaviours and contributing factors to injuries. Environmental (sidewalk riding, parking issues, road surface conditions) and vehicle factors (brake failure, stability) that contribute to crashes are also evidenced. Considering micromobility as a whole, a comparison between traditional bicycles, e-bikes and e-kick scooters in field trials can be performed about kinematic parameters and performances: Dozza et al. [12] highlight the differences in manoeuvrability and in the stability of these vehicles; moreover, e-kick scooters perform differently than traditional bicycles in terms of braking and steering: while a bicycle may be easier to brake, an e-kick scooter is easier to steer. To design proper shock absorbing components and limit stresses to the structure [13], Ventura et al. [14] experimentally assessed the vibrational behaviour of the vehicle-rider system deriving from the differences in wheel size and the centre of gravity position for e-bikes and e-kick scooters. The positive effect of e-kick scooter related to urban mobility conundrums of traffic and air pollution are explored in a study about ten cities in the USA, Europe, and Australia/New Zealand before and after the e-kick scooter rental introduction [15]. The paper concludes that it is prudent for urban planners to introduce policies regarding the integration of various types of vehicles as maximum speeds, mandatory use of PLEV infrastructure, and dedicated parking. Moreover, it notes that the negative public opinion influences the transformation of transport systems.

With the increasing number of microvehicles on the roads, there has also been a growing concern for road safety and the risks associated with their use. Accidents involving PLEVs are increasingly common in cities worldwide and protection of riders is becoming important as that of other vulnerable road users [16], [17], [18]. Safety-related issues include hazards introduced in road scenarios, such as sharing road space with all other motor vehicles, the risks that users face during circulation, and challenges related to vehicle and user protection. Because of the peculiar impact dynamics, specific methods have also been proposed for accident reconstruction in the specific cases where PLEVs are involved [19], [20], [21]. To assess the risk of PLEV-related road accidents, it is essential to consider the circumstances surrounding the injuries. A USA study examines data regarding a six-year period from 2014 to 2019 from the National Electronic Injury Surveillance System (NEISS), which offers a national sample of emergency room visits in the United States [22]. Information regarding injury circumstances was extracted from narrative microvehicle users' comment lines. The most frequent type of road accident was PLEV against motor vehicle in an urban street; road surface conditions prove to be relevant for accident occurrence and stability. An applied logistic regression model shows that PLEV users who crashed with motor vehicles are about 150% more likely to be hospitalized and 30% more likely to suffer a head injury. In particular, the misuse of helmet appears as one of the main contributors to PLEV users' injuries [16], [23]. Hamzani et al. [24] determined that helmet usage among e-bike riders in Finland was 17.7%, being associated with a lower rate of head injuries and bone fractures (18.2% if worn, 37.3% otherwise). Additionally, based on the China In-depth Accident Study database, Hu et al. [25] determined that e-bike collision speed is not significantly associated with the risk of severe and fatal injuries in side impacts with a passenger car; rather, fatality risk for a car speed of 30, 50, 60, 80 km/h reaches respectively 2.9%, 23%, 50%, 90%. Age of the e-bike rider is also a relevant factor on injury severity [16], [25], [26]. Focusing on differences among microvehicles, Verstappen et al. [27] evidenced no substantial variation in Injury Severity Scores (ISS) for e-bike and traditional bike riders (confirmed by Qian et al. [28] and Westerhuis et al. [29]). Hamzani et al. [30] evidenced that no significant association exists between the probability of hospitalization and the use of an e-bike or an e-kick scooter; however, Arbel et al. [31] highlighted that e-bike riders are more likely to sustain maxillofacial fractures than e-kick scooter riders, indicating a higher severity of injuries for e-bike accidents.

The previously reported literature data are precious research tools: in general, the accurate number of road accidents involving PLEVs is not easily attainable due to various reasons. For instance, some countries do not have a centralized database specifically dedicated to e-kick scooter accidents, or accidents may not be categorized with details regarding the type of vehicle involved. Nevertheless, this is a key element for proposing efficient strategies to increase the safety of specific PLEV user categories [17]. The articles with e-kick scooter data reference to non-conventional databases, such as in Yang et al. [32], who considered searching and analysing massive media reports to construct crash datasets covering about 40 states in the USA. Similarly, in Cicchino et al. [33], interviews were conducted with 105 adults who were injured while riding e-kick scooters and presented to an emergency department in Washington, DC, during 2019. The second work's conclusions are that e-kick scooter riders injured on the road were about twice as likely as those injured elsewhere to sustain injuries with AIS (Abbreviated Injury Scale) equal to 2.

Based on these highlights, it is possible to define three levels of safety for PLEV users: at the vehicle level, at the circulation level, and at the user level. At the vehicle level, safety depends on stability, robustness, manoeuvrability of the PLEV as well as other safety equipment (e.g., lighting and braking systems). At the circulation level, sharing road space with other road users, such as motorists, cyclists, and pedestrians, presents unique challenges for microvehicle users, leading to possible conflicts with those road users; analysis of safety from this perspective involves the in-depth investigation of root causes of the accidents (in terms of impact frequency) and also the associated riders' injuries to propose efficient countermeasures. At the user level, the focus is on PLEV user perception of safety, e.g., involving behaviours related to helmet usage, knowledge of traffic rules, responsible vehicle operation, riding under the influence of alcohol, or becoming distracted while riding. Additionally, lack of experience and competence in using microvehicles can increase the risk of accidents and injuries for users [34]. To address these challenges and improve the safety of PLEVs, a comprehensive approach is needed, including uniform regulations, enhanced safety standards, dedicated infrastructure, and user awareness campaigns. So far, there is no uniform worldwide regulation on the use of PLEVs. More specifically, the Europe-wide regulation of PLEVs is also heterogeneous so that some countries classify the relatively new vehicles as equivalent to bicycles or pedestrians, while other countries do not allow them or are pushing ahead the development at a national level. For the development of new PLEVs integrating the most modern safety criteria already at the conceptual stage, it is therefore necessary to:

• identify circulation-relevant regulations, for marketing the vehicle in the widest possible number of countries and with the most effective equipment;
• analyze accident-related aspects in detail so that criticalities at the vehicle level can be overcome by targeted policy and road design strategies;
• determine users' needs for enhancing their riding experience and favour more cautious behaviours.

The main objective of the present work is to propose a holistic approach for determining a novel PLEV concept that includes all relevant features for vehicle safety maximization, specifically in the form of an e-kick scooter. To this aim, regulations applying to PLEVs in European countries are first critically analysed. Triggering factors and contexts of PLEV-related accidents are then investigated, also accounting for the riding behaviour and experience of PLEV users involved and the influence of vehicle design on accident initiation. Surveys are also performed to identify the perception of users with respect to safety when riding e-kick scooters.

## 2 Overall categorization schemes for PLEVs

Since November 2019, micromobility vehicles can be categorised according to the international standard of the Society of Automotive Engineers (SAE). Vehicles defined according to SAE J3194 (SAE International) must be partially or fully powered and must not exceed a maximum curb weight of 500 lb (227 kg), a maximum vehicle width of 5 ft (1.5 m) and a maximum design speed of 30 mph (48 km/h). These vehicles are classified according to the vehicle categories shown in Table 1. The more detailed classification of these vehicles is based on defined codes regarding the curb weight, the vehicle width, the maximum design speed and the energy source. For example, an electrically powered scooter with a curb weight of 40 lb (18.14 kg), a width of 2 ft (60.1 cm) and a speed of 18 mph (29 km/h) would be coded WT1/WD1/SP2/E Powered Standing Scooter. In spite of the standard, international vehicle classification systems differ widely with regard to micromobility. As a result, the International Transport Forum^2^ proposed uniform definition and categorisation of micromobility based on the introduced SAE J3194 standard. The categorisation is also based on the maximum design speed and the curb weight of the vehicle. Type A and B of micromobility vehicles include both vehicles driven by muscle power, such as bicycles, and electrically driven vehicles whose electric drive switches off at 25 km/h. Bicycles, e-bikes, e-kick scooters, Segways and self-balancing electrically powered vehicles would be included in this category. The separation between vehicles with a maximum design speed above or below 25 km/h is based on the frequent exclusion from the use of cycle paths and the extended safety regulations above this speed. The limitation of the curb weight to a maximum of 350 kg is based on the correlation of the kinetic energy of a vehicle and the risk of serious to fatal injuries [35]. Due to the defined limitations in terms of weight and speed, the kinetic energy of the vehicles is limited to 27 kJ, which corresponds to one hundredth of the kinetic energy of a compact vehicle at top speed.

**Table 1:** Classification system for micromobility vehicles according to SAE J3194.

Name	Code	Description
**Curb weight**		
Ultra lightweight	WT1	curb weight ≤ 50 lb (23 kg)
Lightweight	WT2	50 lb (23 kg) < curb weight ≤ 100 lb (45 kg)
Midweight	WT3	100 lb (45 kg) < curb weight ≤ 200 lb (91 kg)
Midweight Plus	WT4	200 lb (91 kg) < curb weight ≤ 500 lb (227 kg)
**Vehicle width**		
Standard-width	WD1	Vehicle width ≤3 ft (0.9 m)
Wide	WD2	3 ft (0.9 m) < vehicle width ≤4 ft (1.2 m)
Extra-Wide	WD3	4 ft (1.2 m) < vehicle width ≤ 5 ft (1.5 m)
**Top speed**		
Ultra low-speed	SP1	Top speed ≤ 8 mph (13 km/h)
Low-speed	SP2	8 mph (13 km/h) < top speed ≤ 20 mph (32 km/h)
Medium-speed	SP3	20 mph (32 km/h) < top speed ≤ 30 mph (48 km/h)
**Power source**		
Electric	E	Powered by an electric motor
Combustion	C	Powered by an internal combustion engine

## 3 Safety at the vehicle level - regulatory situation in European countries focusing on e-kick scooters

Rules for circulation of a vehicle in a country are typically based on past experiences and evidences, aiming at the limitation of problems related to previously unregulated driving/riding aspects. For this reason, the analysis of current regulations in Europe is beneficial for deriving safety criteria that can be directly included in PLEVs during their initial design phase. So far, there is no uniform regulation for the use of PLEVs throughout Europe. Some countries put the relatively new vehicles on an equal level with bicycles, while other countries do not allow them or are pushing ahead the development on their own. Being them the newest vehicles to be admitted to circulate in all European countries, the situation of e-kick scooters is particularly inhomogeneous and critical. In the present Section, various regulations regarding e-kick scooters of selected countries are compiled based on their applicable legal texts. For the development of an e-kick scooter that can be driven in all European countries, the individual technical parameters must be designed to comply with the most strict regulations in Europe. A tabular overview of the regulations is provided in Appendix A, Table 2. All European countries have been considered, but only European countries that apply a specific limitation for circulation are evidenced in Table 2. The strictest regulations from a specific perspective are identified by underlined text.

According to the research, the regulations of the German legislation - the eKFV - have to be followed in most cases. The curb weight for a new vehicle should be based on Danish legislation since the limit for the maximum curb weight including battery is the lowest among all analysed countries (25 kg). The nominal continuous power must be limited to 250 Watts in Norway, Portugal, Sweden, Luxembourg, Slovakia. Regarding the maximum speed, the limit of 20 km/h should be considered. In the Scandinavian countries, there is usually an extra requirement regarding lighting. Front and rear lights must be designed in such a way that their light is still clearly visible at a distance of 300 meters. According to German case law, the use of white light applies to front lighting. The rear lighting must be provided with at least one red rear light and one red non-triangular reflector of category Z. The side markings have to be on both sides with yellow reflectors or with retroreflective white stripes on the hubs or rims of the front and rear wheel. Regarding direction indicators, as of March 2024 regulations are evolving, like in the case of Germany after the evaluation of the eKFV in 2022, in which a mandatory installation of direction indicators is proposed as a recommendation.^3^ From 2023, Italy permits the circulation of e-kick scooters only if equipped with direction indicators. Considering these highlights and the importance this major theme is gaining in Europe, we recommend to install direction indicators in e-kick scooters that are newly introduced in the market. In addition, there are dimensional requirements and limits on both the minimum and maximum mounting heights that must be observed. Considering the German regulation, e-kick scooters must be also equipped with a vehicle identification number, a factory plate, and a holder for the insurance plate; CE-certification is still required. The vehicle should not be longer than 1,000 mm (Spain) and not wider than 600 mm (Spain). The length of the handlebar must be at least 700 mm (Germany). Following the German regulation, the e-kick scooter must have two independent brakes which are effective up to the maximum speed (20 km/h), can brake the vehicle to a standstill, achieve deceleration values of at least 3.5 $m/s^{2}$ and achieve a minimum deceleration of 44% of the total braking effect in case of failure of one brake. The equipment of at least one brightly sounding bell as a sound signal is prescribed in all countries, also based on the requirements from DIN EN 17128 regarding requirements and test methods for PLEVs. The vehicle shall also comply with the safety requirements for electromagnetic compatibility (Regulation No 10 UNECE), the safety requirements for the battery (DIN EN 15194:2018-11) and the measures for protection against tampering (DIN EN 15194:2018-11). In addition, the vehicle must be protected both against direct contact with all live components and against unintentional adjustment of all controls. The vehicle must be such that the control for regulating the engine power automatically returns to the zero position within one second when released. In both Germany and the UK, e-kick scooters must pass several driving dynamics tests to be approved for public road use.

Based on the information in Table 2, it is derived that German regulations cover all possible aspects of circulation apart from the helmet obligation, with the strictest requirements from the standpoints of deceleration and direction indicators. In general, there is no specific country that has the strictest regulations for all circulation-related requirements. However, for effective implementation across Europe, it is advisable to identify the most effective regulations from each country to create a cohesive and standardized framework.

Regulations in the EU also apply to the user's obligations while riding. Even if these are not specifically related to the vehicle itself, it is worth mentioning them to clarify the context of vehicle circulation for the remainder of the work. These aspects are included in Table 3. For a better understanding of the “Lane Usage” section, “Road Lane” describes the area on which motor vehicles participate in traffic, “Bicycle Facility” describes the area of traffic that is specifically intended for cyclists, and “Pavement” describes areas intended for pedestrians.

## 4 Safety at the circulation level - analysis of traffic accidents involving PLEVs

The application of strict regulations for the circulation of PLEVs is not sufficient to avoid the occurrence of road impacts. For this reason, it is crucial to analyse the nature and the outcomes of PLEV-related crashes to propose efficient countermeasures to limit their frequency and potential of provoking injuries. To obtain a comprehensive overview of the number of road crashes involving PLEVs at a European level is challenging because some countries lack a specific category for PLEVs (Table 2), making it difficult to provide accurate figures. The ability to calculate the number of injuries and fatalities in road crashes is also impeded by limited data reporting. If PLEV crashes are identifiable, they are prone to being underreported, a common occurrence for all accidents without the involvement of a motor vehicle. Moreover, there is scarce data available regarding the travel or the distance covered by PLEVs or the duration for which users operate these vehicles. This lack of information makes it challenging to quantify crash risks accurately for this type of vehicle. Considering this, retrospective hospital studies have been performed, examining the records of patients involved in crashes so that the behaviour of PLEV users can be considered for road safety assessments.

In the present Section, data are reported about the crashes, injuries, and fatalities for PLEV-related crashes in several European countries for which 2021 statistics are fully available. The analysis covers the following countries: Austria, Finland, France, Germany, Iceland, Israel, Italy, Lithuania, Netherlands, Poland, Portugal, Slovenia, Spain, Switzerland, and the UK, providing a comprehensive statistical investigation. Subsequently, an investigation of German data is presented, aiming to provide an extensive overview of the following aspects that cannot be derived from national statistics:

• Critical situations involving PLEVs in urban areas;
• Causes and contributing factors to PLEV-related accidents;
• Riding behaviour and experience of PLEV riders involved in accidents;
• Influence of vehicle design on accident initiation.

### 4.1 National statistics in EU regarding PLEV injuries and fatalities

**Figure 1:**
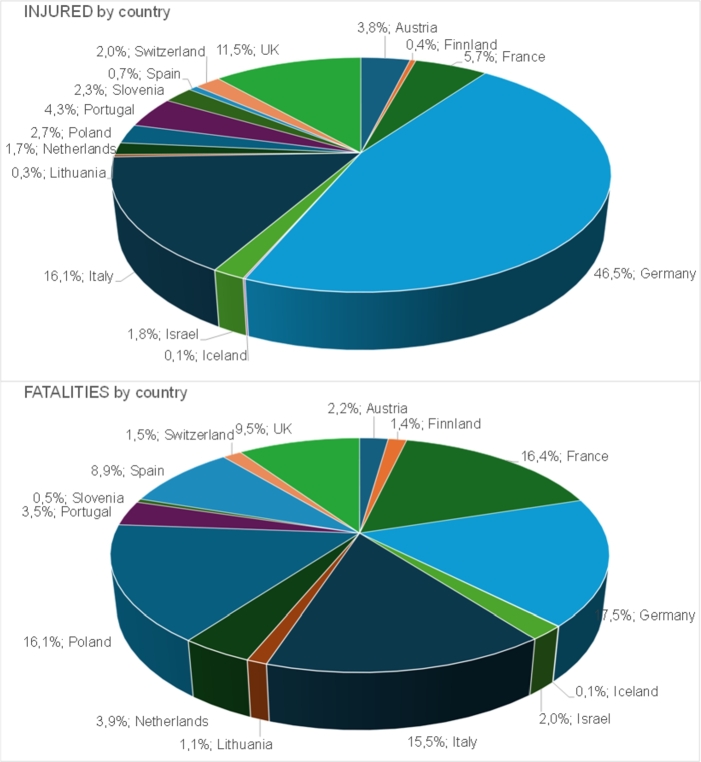
Distribution of injuries and fatalities in considered countries for all types of users (PLEVs, human-powered bikes, other types of vehicles and pedestrians).

This Section explores data related to road accidents by country, with particular attention to injured individuals and fatalities, segmented by the type of vehicle involved. This analysis enables one to draw relevant conclusions regarding accident trends and to identify critical areas to focus efforts on improving road safety in Europe. This allows more accurate comparisons to be made with other data, for example with the GIDAS data (see Section 4.2). The total number of individuals involved in 2020 in accidents is 1.002.843 (injuries and fatalities), distributed across the following countries: Austria, Finland, France, Germany, Iceland, Israel, Italy, Lithuania, Netherlands, Poland, Portugal, Slovenia, Spain, Switzerland, UK. Out of all injured data, 168.290 involve bicycles or PLEV users, while the remaining 819.054 involve other road users. Additionally, 1.952 fatalities have been reported in accidents involving human-powered bicycles or PLEV users, compared to 13.547 fatalities of other road users. The percentages of each country are shown in the charts in Fig. 1. Germany has by far the highest number of injuries for all road users, while France has the highest number of fatalities. The percentage of PLEV users and cyclists among the road users involved in accidents is 16.98%. This indicates a significant presence of PLEV riders and cyclists (users of human-powered bicycles) in proportion to the total number of people involved in road accidents in these countries. The percentage of PLEV users and cyclists among the injured persons is similar, i.e., 17.04%. This suggests that, compared with the total number of injuries, the share of injuries among PLEV users and cyclists is almost equal to their overall involvement in accidents. The total number of deaths in road accidents is 15.499, and the percentage of deaths among PLEV and cyclists is 12.6%. Although PLEV users and cyclists represent a significant proportion of injuries, the percentage of fatalities among them is relatively lower.

**Figure 2:**
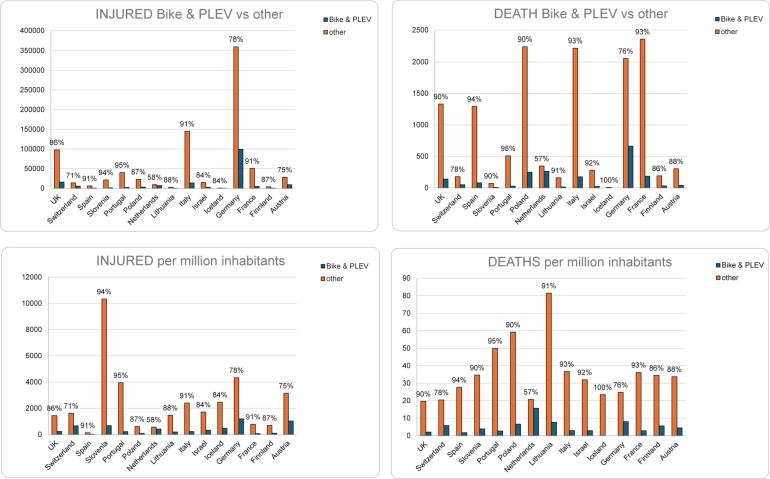
Distribution of injuries and fatalities of users in considered countries (users of bike and PLEVs versus other users).

**Figure 3:**
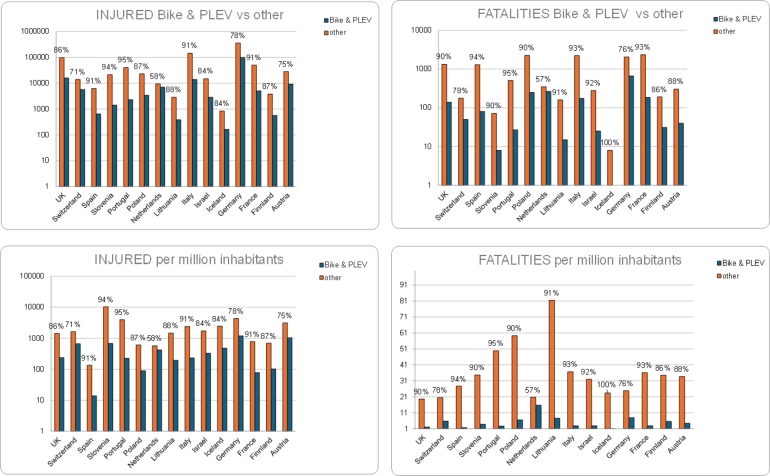
Distribution of injuries and fatalities of users in considered countries (users of bike and PLEVs versus other users), presented on a logarithmic scale.

The substantial presence of PLEV and cyclists among people involved in accidents and injured suggests the need for specific safety measures for these road users in all these countries. This could include awareness campaigns, infrastructure improvements, and specific regulations to ensure the safety of PLEV users and cyclists. For a more comprehensive understanding, we performed a more in-depth analysis, considering details such as the root causes of accidents, road conditions, and other factors that could guide targeted interventions to improve road safety in each country. As for regards the distribution of injuries and fatalities in the various countries, national statistics are shown in Fig. 2 for PLEVs and bike users versus other road users. France has the highest number of fatalities for the other types of road users. Germany has the highest number of injuries for the other types of road users but, most importantly, also the highest number of occurrences for both injuries and fatalities of PLEV and bike users. Iceland has the lowest number of occurrences for both injuries and fatalities of PLEV and bike users; in particular, no fatality is observed in this country. Since the figures are undoubtedly affected by the difference in the population among countries, the number of injuries and fatalities have been expressed per million inhabitants in Fig. 3. As it can be seen, Germany has the highest number of injuries per million inhabitants related to PLEVs and bikes while Spain has the lowest. The Netherlands has the highest number of fatalities per million inhabitants related to PLEVs and bikes. This may be justified considering that a high number of trips in the Netherlands are made by bikes (28%^4^); while it may be relevant to consider the number of kilometres travelled per year by the population, these data are not directly available from reports at a national level. A more detailed analysis to determine the risk of injury and fatality for bike and PLEV users can be performed if the single country statistics are available in terms of kilometres travelled by these types of vehicles.

**Figure 4:**
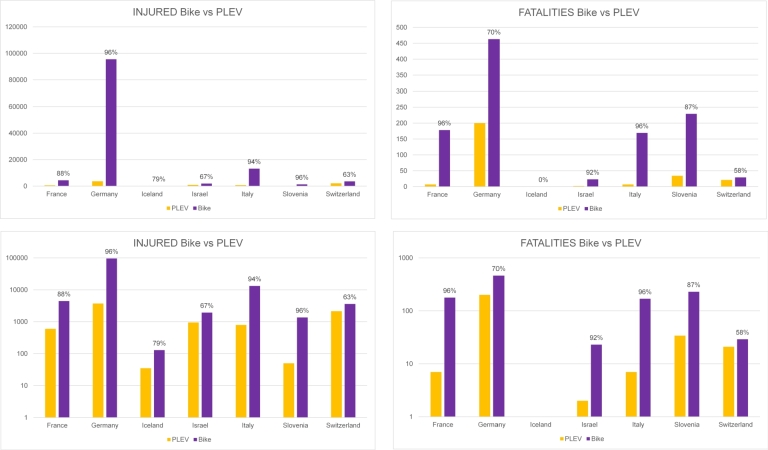
Distribution of injuries and fatalities of users in countries where PLEVs and human-powered bikes are distinctly categorized, presented on a linear scale (above) and on a logarithmic scale (below).

In a subsequent analysis, the data for PLEVs is disaggregated for the following countries where they are separated from human-powered bikes: France, Germany, Iceland, Israel, Italy, Slovenia, Switzerland. This information is crucial to determine whether PLEVs are significantly less safe than human-powered bikes. Since the figures related to PLEVs are low, a logarithmic scale visualization of data is also reported. As can be seen in Fig. 4, only Switzerland and Israel have a large proportion of crashes with involvement of PLEVs compared to human-powered bikes (higher than 30%). Regarding the number of fatalities, one can see that in Germany the percentage of fatalities of PLEV users is high compared to the associated share of injuries, and the same applies to Switzerland. National statistics highlight that the electric alternative may actually pose additional threats compared to the use of a traditional bike in terms of involvement in road impacts, because of their higher tendency in being associated with fatalities rather than injuries.

Based on the previous results, it can be derived that Germany has a very high share of PLEV accidents, injuries, and fatalities among all the analysed EU countries. Moreover, the dedicated categorization and distinction of PLEVs in the German dataset might contribute to a more granular understanding of the specific characteristics and trends related to this mode of transportation. For this reason, a thorough examination of German data is performed to evidence specific critical aspects that can undermine the safety of PLEVs.

### 4.2 Detailed data from the German-In-Depth Accident Study (GIDAS)

Within the scope of the in-depth crash investigation, the findings from the police data are specified with regard to the types and severity of injuries that occur, the use of protective clothing or its potential. This is based on GIDAS data, a cooperative traffic crash research project of the Federal Highway Research Institute and the German Research Association for Automotive Technology. Due to the specific type of use and the emerging typical accident constellations (many solo accidents, frequently drunk drivers), it is to be expected that the number of unrecorded accidents with personal injury by the police is very high. As long as no serious injuries occur and no third party is harmed, it is assumed that the police are rarely notified. In the case of very minor injuries (e.g. abrasions, bruises), these accidents - analogous to cyclists with minor injuries who have fallen only - can hardly be recorded statistically. This aspect is therefore addressed in the survey by questions about accident history.

The evaluations carried out with the official road accident statistics of the Federal Statistical Office of Germany are evaluations of accidents with personal injury involving PLEVs for the period from January to December 2020 and for the period from January to December 2021. In the year 2020, 1,955 accidents with PLEV involvement are recorded; in 2021, more than twice as many accidents with PLEV involvement are registered; in total 5,079 accidents are recorded.

The detailed aspects on accidents with PLEVs are elaborated, about:

• Usual types of accidents;
• Location of accidents;
• Time of accidents (day of week, time of day);
• Causes of accidents;
• Injury severity (official, Maximum Abbreviated Injury Scale (MAIS));
• Typical/frequent injuries;
• Use of protective clothing;
• Derivation of potentials for protective clothing;
• Indications of misconduct (e.g., use of the PLEV by two or more persons);
• Accident mechanisms as a function of driver experience.

The study supplements findings from a comprehensive investigation of hospitalized PLEV users with an analysis of accidents involving PLEV recorded in GIDAS. These accidents, a subset of those reported by the police, cannot be used to estimate unreported cases. The GIDAS dataset provides detailed information, including photos and accident reconstructions, allowing analysis of individual cases.

Moreover, the police-collected accident data vary in detail, often containing additional information like helmet usage and unauthorized passengers on PLEVs. To delve deeper into these details, individual accident data from Saxony police are examined and stored in the “electronic accident type cards” (EUSKa) database. EUSKa data are analyzed for comprehensive accident monitoring, allowing detailed examination of accidents and specific characteristics. This includes accidents resulting in personal injury or property damage into PLEV accidents, in the Saxon. However, determining helmet usage rates is challenging due to limited recording in EUSKa data, hampering conclusions about PLEV users' helmet-wearing behaviour.

#### 4.2.1 Accident parameters

Looking at the seasons in the GIDAS data at a meteorological level, comparatively few accidents involving PLEVs occurred especially in spring (March-May – one accident) and winter (December-February – four accidents). Similar to the evaluation of the EUSKa data, most accidents occurred in autumn (September-November – 20 accidents) and summer (June-August – 15 accidents), although the EUSKa data shows slightly more accidents in summer than in autumn. Moreover, the least frequent time period is from 08:00 am-11:59 am. The largest number of accidents occurred between 00:00 am-03:59 am. 24 of the 40 accidents therefore occurred at dusk, at night, and in the early morning hours (08:00 pm-07:59 am). If the corresponding GIDAS variable on light conditions is evaluated, 20 accidents occur at night and two at dawn. However, this variable is not coded for four accidents and is therefore unknown. 14 accidents occurred during the day.

#### 4.2.2 Accident severity and injury analysis

According to the official injury severity, none of the accidents in the GIDAS database resulted in a fatal injury. In 26 accidents, and thus the majority, the maximum official injury severity was light. However, 13 accidents resulted in at least one serious injury. In one accident, the injury severity is unknown. In only four cases the collision opponent was slightly injured, and in 11 cases he/she was not injured. Thus, the higher injury severity is mostly on the side of the PLEV user. Of the total of 46 people who used a PLEV, only five passengers were uninjured, 26 were slightly injured and 13 were seriously injured. The injury severity is unknown for two passengers. Despite the low number of entries, these data are extremely difficult to retrieve and are precious to get more detailed information on accident causes and injury mechanisms.

**Figure 5:**
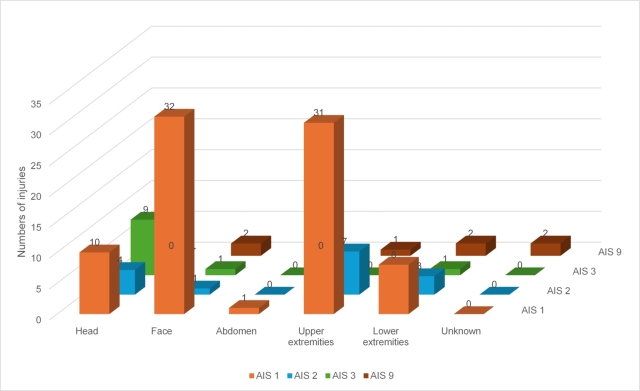
Distribution of injury severity according to body region (code AIS), data source: GIDAS.

A total of 115 individual injuries are available in the GIDAS database for these 39 slightly or severely injured PLEV users (AIS codes according to the 2015 revision). The distribution of AIS for the various injuries according to the body region is reported in Fig. 5.

With a total of 39 individual injuries, injuries to the upper extremities occurred most frequently, closely followed by 36 injuries to the face. Furthermore, head injuries were also frequently recorded (23 times). 14 injuries occurred to the lower extremities and only once was the abdomen affected. Cervical, thoracic and spinal injuries have not yet been recorded in the GIDAS database. However, this result highlights that protecting the upper extremities is the priority for these types of vehicles.

**Figure 6:**
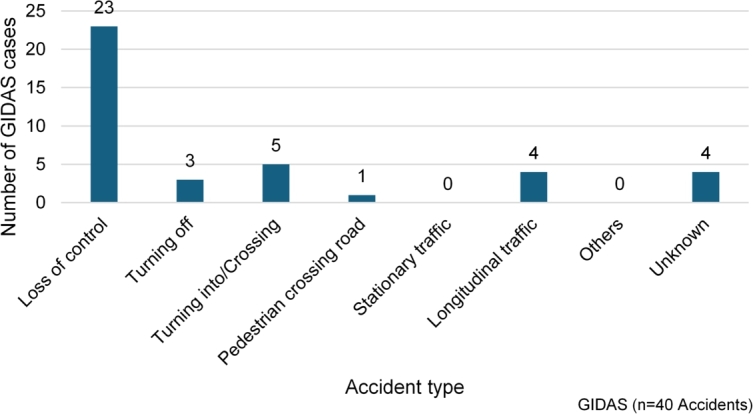
PLEV accidents distribution, data source: GIDAS.

Similar to the EUSKa evaluation, loss of control accidents also occur most frequently in GIDAS. Of the 40 accidents in this database, the accident type is known for 36, and 23 of these can be assigned to loss of control accidents. The other accident types have occurred less frequently, accidents involving stationary vehicles and other accidents have not occurred at all so far. The distribution of PLEV accidents by type of accident is shown in Fig. 6. These figures highlight that stability is the most relevant problem during PLEV riding and countermeasures from this perspective should be considered to increase the user's safety.

#### 4.2.3 Main causer, causes of accidents and influence of alcohol

An evaluation of the main accident causer is meaningful for accidents with at least 2 participants. The majority of accidents were single-vehicle accidents; as already mentioned, a second party was involved in 14 accidents only. In eight of these accidents, the collision counterpart was the main causer of the accident and thus in six accidents it was the PLEV user himself. Among these six accidents, there was also one PLEV-PLEV collision. For the evaluation of the causes of accidents, partial fault is also considered, which is why the sum of all the following data differs from the sum of all participants. Unknown characteristics were also neglected here. The (first) cause of the crash of the collision opponent is distributed among the different categories as:

1. No cause: 4 times;
2. Turning, reversing, entering and starting: 3 times;
3. Right of way, priority: 2 times;
4. Wrong behaviour of pedestrians / Wrong behaviour when crossing the carriageway: 2 times;
5. Driver failure, road use: 1 time.

The first cause of accidents of the PLEV users is distributed as follows. It should be mentioned here that the influence of alcohol is often coded as a second cause and therefore a separate analysis follows.

1. Other causes: 20 times;
2. Driver failure, road use: 7 times;
3. No cause: 5 times;
4. Right of way, priority: 3 times;
5. Wrong behaviour towards pedestrians: 1 time.

**Figure 7:**
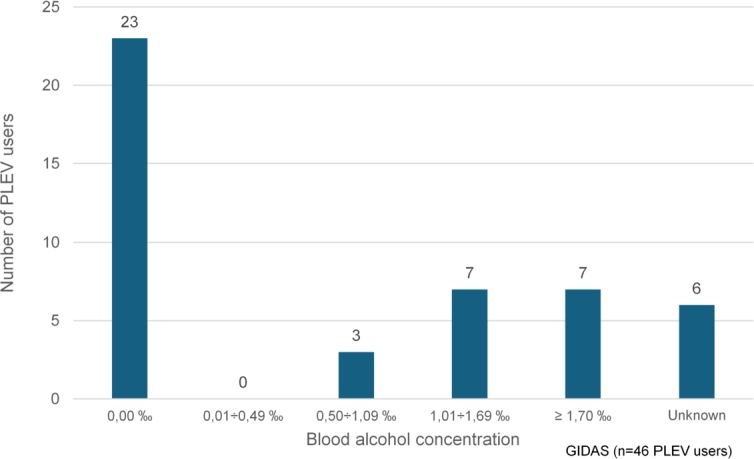
Blood alcohol concentration of PLEV users involved in accidents, data source: GIDAS.

The analysis of alcohol influence is carried out for the 46 PLEV passengers, of which six persons have this variable unknown in GIDAS. Of the remaining 40 persons, 23 drove without being under the influence of alcohol, i.e. 0.0. For this evaluation, the blood sample was examined. None of the intoxicated users had an alcohol level between 0.0 and 0.49. The remaining 17 persons exceeded the legally permitted blood alcohol content of 0.5 in a crash with a PLEV. This means that 42.5% of the PLEV users in GIDAS who were involved in a crash and had a blood sample taken were driving under the influence of alcohol. One drink-driving PLEV user left the scene of an accident in breach of duty after a collision with a cyclist. The exact divisions into the different blood alcohol concentration ranges can be seen in Fig. 7.

#### 4.2.4 Infrastructure conditions

Similar to the EUSKa evaluation, most accidents with PLEV involvement occurred on straight carriageways (21 accidents), followed by eight accidents at junctions and five accidents at intersections. Two accidents occurred at driveways. For the remaining four accidents, the location of the accident is unknown. The driver of the PLEV used the cycle path in 12 cases. Driving on the road in accordance with the rules took place in a total of seven accidents. Two accidents occurred when crossing the road. In ten accidents, the pavement was used in violation of the rules, and in another six accidents, the road was used, although a cycle path was available. In most of the GIDAS crashes with PLEV involvement, the road surface was dry at the time of the accident (28 times). In seven cases, the road surface was damp or wet. One accident occurred in hoar frost.

### 4.3 In-situ study of PLEV crashes in Dresden (DE)

For further information regarding the crash from the perspective of the rider, a comprehensive investigation was performed in the Dresden area to augment knowledge derived from police accident data regarding PLEV-related crashes. The study involves collecting and analyzing data from injured PLEV users treated at several hospitals, with a focus on types and severity of injuries, use of protective clothing, and related factors. As for the implementation of the studies, several hospitals have been involved, i.e., University Hospital “Carl Gustav Carus” Dresden (UKD), Hospital Dresden-Friedrichstadt, Hospital Dresden-Neustadt, Diakonissenkrankenhaus Dresden. Data were collected by the medical staff, with patients encouraged to cooperate by responding to a brief questionnaire for in-depth data collection. Information was coded in a database, covering user-specific data, accident details, and injury specifics. The study was led by the medical director of the Surgical Emergency Department at UKD University Hospital Dresden, with the administrative supervision and support from VUFO. VUFO also dealt with data collection and processing. The study content was kept minimal due to time constraints in the emergency room, by a simple, self-explanatory survey format with drop-in or categorical answers. A structured database was created by VUFO, including GPS position of cases. Medical data were classified based on AIS 2015 assessment, including seat, impaired body part, and MAIS value for injury summary. 78 patients could be registered. The current official definitions are used to classify the degree of injury severity (slightly injured – outpatient treatment, seriously injured – inpatient stay, killed – died within 30 days). A total of 45 persons with minor injuries and 25 persons with severe injuries are recorded. For eight persons, the injury severity could not be determined. This corresponds to a ratio of 32% seriously injured and 58% of slightly injured (10% unknown). Accidents were predominantly recorded by PLEV users under the age of 40.

Overall, a large proportion (56 accidents, approximately 72%) of the accidents registered in the special survey are solo accidents. This is in line with data reported in Section 4.2 (23 solo crashes out of 40 in total). For eight cases it is unknown whether other persons were involved in the accident. In 59 cases, the PLEV was ridden in accordance with the rules by only one person. In eleven cases, the vehicle was used to transport passengers. In eight cases, the number of drivers is unknown.

**Figure 8:**
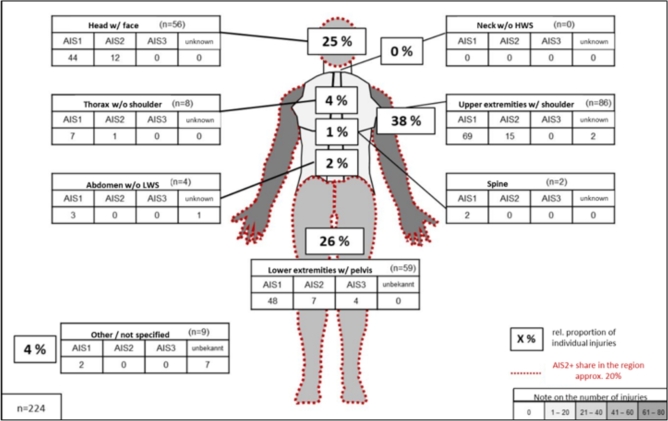
PLEV users by number/type of injuries according to body region and injury severity.

Only two people wore a helmet. 70 people have ridden without a helmet and for another six people the use of helmets is unknown. 224 individual injuries have been coded for 78 PLEV users who had an accident. The distribution of injury severity according to the MAIS shows that most of the injured users suffered minor (MAIS 1, 48%) to moderate (MAIS 2, 34%) injuries. Four persons suffered serious injuries (MAIS 3, 5%). MAIS is unknown for 10 persons (13%). Most individual injuries (Fig. 8) were documented in the upper extremities (including shoulder 38%), followed by the lower extremities region (including pelvis 26%). The third most common injuries were documented in the head and face region (25%).

Among the moderate injuries (AIS 2+), the head and face regions accounted for the highest proportion (21%) followed by the lower (19%) and upper extremities (17%). The most severe injuries with a severity of AIS 3 were all fractures of the lower extremities (three femur fractures and one open tibia fracture). The in-depth examination shows that many injuries are due to defensive movements (e.g. catching with the hands) in case of a fall. In addition, cuts, open wounds and sternal fractures often result from conflicts with one's own vehicle. A solid analysis of injury-causing parts was not possible due to the data retrieval procedure. However, considering the thorax and abdomen AIS, less rigid steering columns could have a positive influence on the risk of injury.

## 5 Safety at the user level - questionnaires and acceptance of current technologies

In addition to the investigation conducted on PLEV accidents in the Dresden area, inquiries were made regarding the personal perception of vehicle safety by users in daily riding. This means exploring factors such as their opinions on protective clothing, perceived vehicle stability, and any specific safety measures they believe are crucial on a PLEV. The approach aims to gain a thorough understanding of users' perceptions and opinions regarding the safety of Personal Light Electric Vehicles (PLEVs) and how this can be ameliorated. This comprehensive summary outlines the driving dynamics and legal requirements governing PLEVs in Germany, covering technical specifications, safety standards, and traffic law compliance. The survey involved 1,501 usual PLEV riders. The users were asked about their subjective perception regarding their behaviour and their safety concerns, including factors such as accident risk, vehicle stability, safety devices. Furthermore, an overview is given here of improvements mentioned with regard tips for updating existing rules, to driving dynamics, uncertainties when riding, features desired by the surveyed users.

**Figure 9:**
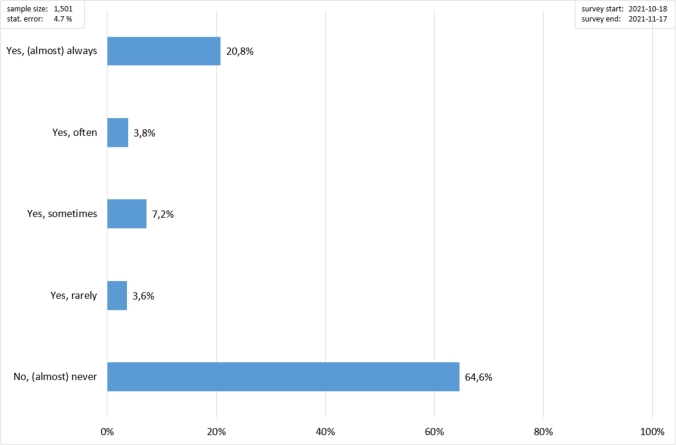
Proportion of PLEV users wearing a helmet.

**Figure 10:**
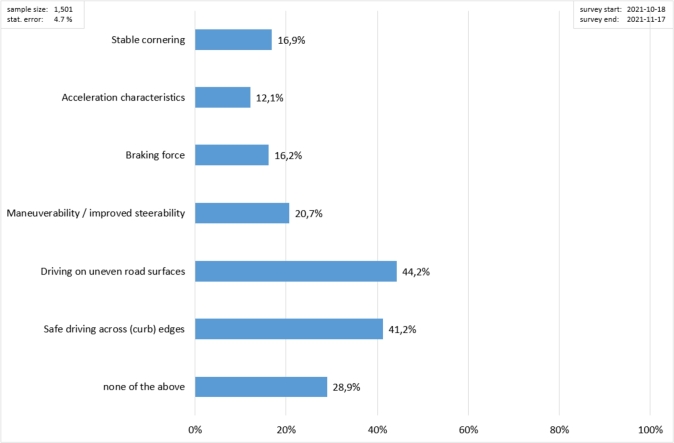
Survey results regarding how the PLEV riding process can be ameliorated in the users' opinion.

First of all, considering the results of the survey in Fig. 9, wearing a helmet is very uncommon for PLEV riders (never wear 64.6%); this is also in line with both the literature [16], [23], [24] and the results in Section 4. Considering Fig. 10, riding on uneven road surfaces and over curb edges with a PLEV is reported to be the major required improvement. In the users' opinion, the less relevant aspects to be enhanced are the acceleration characteristics (both acceleration and braking).

**Figure 11:**
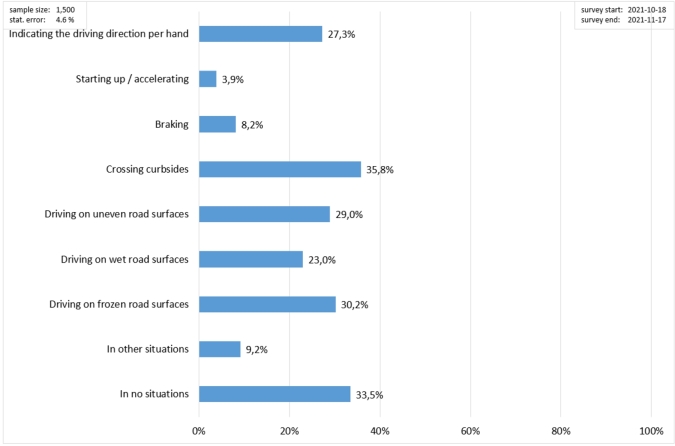
Survey results regarding the situations when the user feels insecure when riding a PLEV.

Based on Fig. 11, the users evidence that crossing curbsides is the most relevant cause of insecurity when riding a PLEV; driving on uneven road surfaces, on wet road surfaces, and frozen road surfaces are also major cause of insecurity (between 20% and 30% of responses). Another significant problem consists of indicating the driving direction by hand, that is relevant for 27.3% of the users. Once again, acceleration and braking are not perceived as a critical matter in PLEV usage (less than 10%).

Based on these highlights, the two most frequently requested improvements (driving on uneven ground and safe riding over edges, Fig. 10) can be met by employing pneumatic tyres with a large diameter and increased ground clearance. Here, too, pneumatic tyres with a large diameter and increased ground clearance are a good solution for the insecurity involved in riding over edges and on uneven surfaces. Another uncertainty exists when indicating the change of direction by hand signal. This uncertainty can be definitely countered by installing direction indicators on the vehicle.

## 6 Derivation of a vehicle safety concept

Focusing on the development of a PLEV in the form of an e-kick scooter, a vehicle safety concept can be derived from the information about the current regulations in Europe, data related to crashes, and the survey by PLEV users. The maximum speed should remain at 20 km/h, considering the speed limit for several countries (Table 2). The braking system should provide two independent braking systems that achieve a minimum deceleration of 3.5 $m/s^{2}$. It should also be possible to operate these two independent braking systems via the handlebars. It is also advisable to install an additional foot brake. This ensures safe braking and allows the users' wishes to be integrated. With pneumatic tyres and large wheel diameters, a more stable ride can be achieved; additionally, these tyres also provide for a safe feeling when driving over edges and uneven surfaces. Large wheel diameters can also increase ground clearance. High ground clearance is likewise helpful for driving over edges safely and for driving on uneven ground. The caster should be selected so that stable straight-ahead driving can be ensured. Stable driving conditions can reduce the number of “loss of control” accidents, in according to Fig. 6.

Furthermore, the following safety devices are especially recommended:

• Lighting devices;
• Installed and electrically operated direction indicators;
• Front/side/rear reflectors;
• Bell.

Lighting devices increase the driver's own perception and at the same time increase the perceptibility of other road users, in particular during nighttime when accidents are more frequent. The installation and electrical operation of direction indicators prevents unstable riding conditions, as the user does not have to take his hands off the handlebar. In addition, the perceptibility by other road users can be increased. A bell on the handlebars can reduce critical situations, as it can provide an acoustic warning by PLEV users. This is especially the case when the traffic area is shared with pedestrians or bicyclists, like in pedestrian areas and on bike paths. Considering the possibility of conflict between microvehicles and pedestrians (also refer to Section 4.2.3), the presence of specific lightweight bumping elements can be envisaged to increase the safety towards all types of road users. This can be achieved for instance by application of specially-designed sandwich structures [36], [37], [38], [39]. In addition, the introduction of a minimum age or the introduction of a “license to ride PLEVs” could increase road safety, as this would provide proof of adequateness to participate in road traffic. However, this could apply also to traditional human-powered bikes, since traditional bicycle users still represent a high share of road crash injuries and casualties.

**Figure 12:**
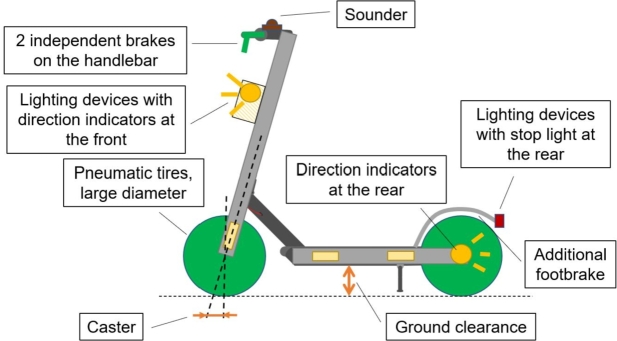
Vehicle safety concept.

To counteract misuse due to passenger transport, weight-dependent control of the motor could be helpful; analogously, a smaller deck could contribute to discouraging the use of the PLEV by more than one person at a time. Table 4 reports an overview of the determined features for a novel vehicle concept, while Fig. 12 graphically depicts a possible microvehicle proposal. All these features will be introduced in a novel vehicle concept and treated more in-depth in future research.

## 7 Conclusions

In this study, we analysed safety of Personal Light Electric Vehicles (PLEVs). The objective was to identify efficient equipment and solutions that can be included in new PLEVs at the design stage, so that users' safety can be maximized; the example of a novel e-kick scooter concept is proposed to demonstrate the applicability of the obtained results. A regulatory review has been initially proposed, revealing a fragmented landscape of European regulations governing PLEVs, especially for e-kick scooters. Harmonization of regulations can create a consistent framework that would enhance safety and facilitate compliance of vehicles to any environment; at the moment, regulations from Germany cover the majority of possible aspects related to vehicle safety, representing a solid basis for the development of innovative vehicles. Through in-depth accident statistics, we have identified that vehicle stability and user awareness are critical factors in preventing accidents. Our findings suggest that manufacturers should prioritize the stability of PLEVs in their design, and that there is a significant need for educational campaigns to raise awareness about the proper use of these vehicles. Finally, we explored user perception related to PLEV riding, which reveals a dichotomy between the appreciation for the convenience these vehicles provide and concerns about their safety. It is evident that while PLEVs are employed for their efficiency and environmental benefits, there is apprehension about their use in spaces shared with other road participants. To address this, a vehicle safety concept has been proposed, integrating all possible aspects that can be derived from regulations, accident analysis, and users' needs.

In conclusion, our research can be seen as a call to action for policymakers, manufacturers, and users alike to collaborate in creating a safer and more sustainable future for urban mobility. The integration of PLEVs into our cities presents a unique opportunity to reshape urban transport, but it must be approached with caution, foresight, and appropriate solutions.

## CRediT authorship contribution statement

**Michelangelo-Santo Gulino:** Writing – original draft, Visualization, Methodology, Conceptualization. **Susanna Papini:** Writing – original draft, Resources, Data curation, Conceptualization. **Giovanni Zonfrillo:** Writing – review & editing, Supervision, Formal analysis. **Peter Miklis:** Writing – original draft, Methodology, Investigation, Formal analysis. **Thomas Unger:** Supervision, Investigation, Formal analysis. **Dario Vangi:** Writing – review & editing, Resources, Project administration, Methodology.

## Declaration of Competing Interest

The authors declare the following financial interests/personal relationships which may be considered as potential competing interests: Dario Vangi reports financial support was provided by 10.13039/501100000780European Commission (Grant Agreement n°101006687). The other authors declare that they have no known competing financial interests or personal relationships that could have appeared to influence the work reported in this paper.

## Figures and Tables

**Table 2:** Summary of regulatory requirements for e-kick scooter characteristics by countries.

**Max curb weight (kg):**	
25	Denmark, Spain, Ireland
55	Germany, United Kingdom

**Speed Limit (km/h):**	

20	Denmark; Germany; Italy; Norway; Spain
25	Austria; Belgium; Czech Republic; Estonia; France;
	Finland; Greece; Iceland; Israel; Latvia; Luxembourg;
	Netherlands; Poland; Portugal; Slovakia; United Kingdom

**Max Power (W):**	

250	Israel; Luxembourg; Ireland, Norway; Portugal; Slovakia;
	Slovenia, Sweden
350	France
400	Netherlands
500	Germany; Italy; Switzerland; United Kingdom
600	Austria
1000	Estonia; Finland, Czech Republic

**Vehicle Dimension:**	

Maximum Vehicle Width (mm)	
600	Spain
700	Denmark; Germany; United Kingdom
800	Estonia
850	Norway
Minimum Handlebar Height (mm):	
700	Denmark


**Vehicle Category:**	
Dedicated category	Austria; Belgium; Estonia; France; Germany; Israel; Latvia;
	Spain; United Kingdom
Bicycles	Czech Republic; Denmark; Greece; Iceland; Italy; Luxembourg;
	Norway; Poland; Portugal; Slovakia; Slovenia; Sweden
Pedestrian and Bicycles	Finland
Light mopeds	Netherlands; Switzerland

**Vehicle mandatory equipments:**	

Direction indicator	Italy
Braking system	Germany: deceleration limits
Front/side/rear reflective markings	All EU
Sounding bell (horn)	All EU

**Table 3:** Summary of users obligations for circulation.

**Minimum Age:**	
12	Austria; France
14	Germany; Latvia; Switzerland;
15	Denmark; Greece; Iceland; Slovakia
16	Israel; Luxembourg; Portugal
18	Italy without license and insurance (14 if both available)

**Helmet Obligation:**	

Austria	Only for persons younger than 12 years
Czech Republic	Only for persons younger than 18 years
Estonia	Only for persons younger than 16 years
Greece	For all
Iceland	Only for persons younger than 16 years
Israel	For all
Sweden	Only for persons younger than 15 years

**Lane Usage:**	

Bicycle facility/Road lane	Austria; Czech Republic; Denmark; France; Germany;
	Israel; Luxembourg; Netherlands; Poland; Portugal;
	Switzerland; United Kingdom
Pavement/Bicycle facility	Finland; Slovenia
Pavement/Bicycle facility/Road lane	Belgium; Estonia; Iceland; Latvia; Norway; Slovakia; Sweden
Road lane	Spain

**Insurance:**	

Insurance	Denmark; France; Germany; Israel; United Kingdom

**Table 4:** Overview of the developed vehicle concept.

Concept	Reason
Maximum speed 20 km/h	Tendency of oscillation at increased speed
Design of the braking system	Ensuring stable braking behaviour
Air tires and large wheel diameters	Stabilization effect due to the gyroscopic force
High degree of ground clearance	Stable driving over obstacels
Large caster	Ensuring stable straight-ahead driving
Safety devices	Safe operation
Safety regulations of Germany	Safe use
Introduction of a minimum age	
or the proof of a “driving license”	Safe use in road traffic
Weight-dependent control of the motor	Prevention of misuse

## Footnotes

1 https://urban-mobility-observatory.transport.ec.europa.eu/resources/case-studies/rise-micromobility_en.

2 “Safe Micromobility”, https://www.itf-oecd.org/safe-micromobility, last accessed 14/05/2024.

3 Bundesanstalt für Straßenwesen, 2022, https://www.bast.de/DE/Publikationen/Berichte/unterreihe-f/2019-2018/f125.html?nn=1844934, last accessed 14/05/2024.

4 The Netherlands by numbers, https://bicyclenetwork.com.au/newsroom/2021/03/11/the-netherlands-by-numbers/, last accessed 14/05/2024.
